# Efficacy and safety of tongxinluo capsule for angina pectoris of coronary heart disease: an overview of systematic reviews and meta-analysis

**DOI:** 10.3389/fcvm.2024.1229299

**Published:** 2024-02-13

**Authors:** Liuying Li, Peimin Feng, Wenhua Zhou, Biao Luo, Lvyu Deng, Daohui Gan, Xiaohan Wu, Fengya Zhu, Xia Zhou

**Affiliations:** ^1^ Hospital of Chengdu University of Traditional Chinese Medicine, Chengdu, China; ^2^ Traditional Chinese Medicine Department, Zigong First People’s Hospital, Zigong, China

**Keywords:** tongxinluo capsule, angina pectoris, coronary heart disease, overview, traditional Chinese medicine

## Abstract

**Background:**

Tongxinluo capsule (TXLC) is a common drug for treating angina pectoris of coronary heart disease (CHD). In recent years, many systematic reviews (SRs) and meta-analyses (MAs) have reported the efficacy and safety of TXLC for improving angina symptoms in patients with CHD. We aimed to comprehensively evaluate the existing SRs and MAs of TXLC in treating angina pectoris of CHD, summarize the evidence quality, and provide scientific evidence and recommendations.

**Methods:**

We searched seven databases for relevant SRs/MAs published up to 1 June 2023. Two reviewers independently completed the literature retrieval, screening, and data extraction. We used A Measurement Tool to Assess Systematic Reviews 2 (AMSTAR 2) to evaluate the methodological quality, the Risk of Bias in Systematic Reviews (ROBIS) to assess the risk of bias, and the Grading of Recommendations Assessment, Development and Evaluation (GRADE) to determine the strength of the evidence. RevMan 5.3 was used to synthesize data.

**Results:**

We identified 15 SRs/MAs, including 329 RCTs and 33,417 patients. According to the evaluation results of AMSTAR-2, only one SR was of high methodological quality, the others were very low. ROBIS assessment showed that one SR (6.67%) had a low risk, 3 SRs (20%) had an unclear risk, and 11 SRs (73.33%) had a high risk. We assessed 42 outcomes by the GRADE, 10 (23.81%) for moderate-quality evidence, 17 (40.48%) for low-quality evidence, and 15 (35.71%) for very-low-quality evidence. Mate-analysis showed that TXLC combined with conventional western medications improved electrocardiogram efficacy (RR = 1.38, 95% CI: 1.23–1.43, *P* < 0.001) and angina efficacy (OR = 3.58, 95% CI: 3.02–4.24, *P* < 0.001), reduced angina attack frequency (SMD = −0.54, 95% CI: −0.64 to −0.44, *P* < 0.001) and angina duration (SMD = −0.42, 95% CI: −0.57 to −0.28, *P* < 0.001), with general heterogeneity. The pooled results showed that TXLC appears to have some efficacy in improving cardiac function and relieving angina symptoms, but there is limited evidence that it improves cardiovascular event rates, hemorheology, lipids, or hs-CRP. In the assessment of drug safety, TXLC was associated with different degrees of adverse drug reactions.

**Conclusion:**

Based on the evidence, TXLC may be effective as an adjuvant treatment for angina pectoris of CHD. However, the quality of the evidence is low, and the drug's safety must be carefully interpreted. In future studies, high-quality randomized controlled trials are needed to confirm the effectiveness and safety of TXLC.

**Systematic Review Registration:**

http://www.crd.york.ac.uk/PROSPERO/, identifier (CRD42022365372).

## 1 Introduction

According to the World Health Organization reported, the number of people with cardiovascular disease (CVD) worldwide increased from 271 million in 1990 to 523 million in 2019. The number of CVD patients in China is about 330 million, including 11.39 million coronary heart disease (CHD) patients (1). CHD is the most common type of CVD and the leading cause of death globally, bringing a heavy economic burden and health threat to the world's population (2). Angina pectoris of CHD is a common clinical disease with high mortality in the acute stage.

Conventional treatment for angina pectoris of coronary heart disease mainly includes antiplatelet aggregation, plaque stabilization, risk factor reduction, antianginal drugs, and revascularization. However, some limitations exist, such as clinical resistance to drugs and long-term side effects (3–5). The individual efficacy of current treatment regimens varies greatly, and patient compliance could be better, which makes it difficult to obtain satisfactory clinical efficacy. Therefore, finding potential ways to relieve angina pectoris of CHD is necessary. TXLC is a traditional Chinese medicine preparation developed by Shijiazhuang Yiling Pharmaceutical Co., Ltd. for treating angina pectoris and chest tightness caused by myocardial ischemia. It mainly comprises ginseng, leech, scorpion, centipede, chuanxiong, and borneol. It has the effect of benefiting qi and promoting blood circulation, dreading collaterals, and relieving pain. Clinically, TXLC plays a positive role in protecting the vascular endothelium, dilating the coronary arteries, enhancing cardiac contractility, improving myocardial ischemia, lowering blood lipids, stabilizing plaques, alleviating angina pectoris and preventing coronary embolism and myocardial infarction (6, 7).

TXLC is widely used in China and has been recommended by multiple guidelines and expert consensuses for treating angina pectoris. A meta-analysis showed that TXLC has an excellent secondary prevention effect on angina pectoris of CHD (8) and has beneficial effects on preventing adverse cardiovascular events (9). However, during treatment, TXLC can also cause gastrointestinal reactions, limb weakness, dizziness, headache, and other adverse drug reactions (10). Because of this, it is necessary to comprehensively evaluate the efficacy and safety of TXLC in treating angina pectoris of CHD to provide better evidence.

## 2 Methods

### 2.1 Search strategy

We searched three international databases (PubMed, Cochrane Library, and Embase) and four Chinese databases (CNKI, SinoMed, Wanfang, and VIP) to identify eligible SRs/MAs published up to 1 June 2023 without language restriction.

Taking the PubMed database as an example, the specific retrieval formulas are as follows. In addition, we manually searched the list of references in the included SRs. The details are shown in the Supplementary Material of the search strategy.

*#1 ((((((((Angina Pectoris [MeSH Terms]) OR (Coronary Disease [MeSH Terms])) OR (Coronary heart disease[Text Word])) OR (CHD [Text Word])) OR (Coronary atherosclerotic heart disease [Text Word])) OR (coronary atherosclerotic cardiopathy [Text Word])) OR (angina [Text Word])) OR (stenocardia [Text Word])) OR (angor pectoris [Text Word])*
*#2 (((((Tong-xin-luo) OR (Tong xin luo)) OR (tong xin luo)) OR (Tongxinluo)) OR (tongxinluo)) OR (tongxinluo capsule) [[All Fields]*
*#3 ((systematic review [Text Word]) OR (systematic evaluation [Text Word])) OR (meta-analysis [Text Word])*
*#4 #1 AND #2 AND #3*

### 2.2 Inclusion criteria

We included SRs and MAs based on RCTs of TXLC for angina pectoris of CHD. Subjects were patients with stable/unstable angina pectoris of CHD without restriction on gender, age, and course of the disease. The treatment group included TXLC or TXLC combined with conventional therapy. The control group only received conventional therapy. The primary outcome measures were ECG improvement and angina symptom relief. Secondary outcome measures included the incidence of cardiovascular events, frequency of angina attack, duration of angina, hemorheology, lipid levels, hS-CRP, and adverse reactions. At least one direct result for cardiac conditions was reported in each SR.

### 2.3 Exclusion criteria

We excluded an SR if it met any of the following criteria: (a) SRs or MAs not based on RCTs; (b) TXLC was not the only treatment or adjuvant treatment in the experimental group; (c) repeat publications; (d) unable to obtain the full text or incomplete data presented and (e) other types of research, such as animal experiments, protocols, conference papers, and case reports.

### 2.4 Study selection and data extraction

According to the comprehensive retrieval strategy, two reviewers independently retrieved and screened the literature. The opinion of a third reviewer was sought when there was a disagreement. After identifying eligible studies, two researchers independently extracted relevant data using standardized extraction tables, such as author, publication year, sample size, diagnostic criteria, interventions, outcomes, adverse reactions, conclusions, etc. Two reviewers cross-checked the extracted content; a third reviewer was consulted to resolve discrepancies.

### 2.5 Assessment of SRs

SRs that met the inclusion criteria were independently assessed by two reviewers for the methodological quality of the SRs, the quality of evidence, and the risk of bias.

#### 2.5.1 AMSTAR 2

Two reviewers used A Measurement Tool to Assess Systematic Reviews 2 (AMSTAR 2) (11) to evaluate the methodological quality of SRs. This tool includes 16 items, with items 2, 4, 7, 9, 11, 13, and 15 considered essential. Items 2, 4, 7, 8, and 9 are rated as yes, no, or partially yes. The study quality was judged as high when no or only one non-essential item did not meet the requirements; medium when more than one non-essential item did not meet the requirements; low when any of the essential items did not meet the requirements and very low when more than one essential item did not meet the requirements.

#### 2.5.2 ROBIS

We used the ROBIS tool (12) to assess the risk of bias (RoB) for SRs, including four key areas: (a) study eligibility criteria, (b) study identification and selection, (c) data collection and study appraisal, and (d) synthesis and findings. Finally, we divided the risk level into “low risk,” “high risk,” and “unclear risk.” One person assessed RoB, another checked this assessment, and both reviewers discussed the results. In the case of disagreement, a third party was consulted.

#### 2.5.3 GRADE

Two researchers independently used the Grading of Recommendations Assessment, Development, and Evaluation (GRADE) tool (13) to evaluate the quality of the evidence. The tool includes five aspects: RoB, inconsistency, indirectness, imprecision, and publication bias. We graded the quality of evidence as “high,” “moderate,” “low,” or “very low.” The two reviewers cross-checked the results, and a third reviewer resolved disputes.

### 2.6 Data synthesis and analysis

RevMan5.3 is used for statistical analysis of the data. Relative risk (RR) was used as the statistic for categorical variables. For continuous variables, mean difference (MD) was used as the statistic if the measurement method and units were the same. Standardized mean difference (SMD) was used as the statistic if measured by different methods or with different units, and its 95% confidence interval (CI) was calculated. Heterogeneity was tested using the *I*^2^ quantitative method. If *I*^2 ^≤ 50%, homogeneity was considered good, and Meta-analysis was performed using a fixed-effects model; if *I*^2^ > 50%, statistical heterogeneity between studies was indicated. Meta-analysis was performed using a random-effects model and, if necessary, subgroup or sensitivity analysis. Publication bias was judged using funnel plots.

## 3 Results

### 3.1 Search results

**Figure 1:**
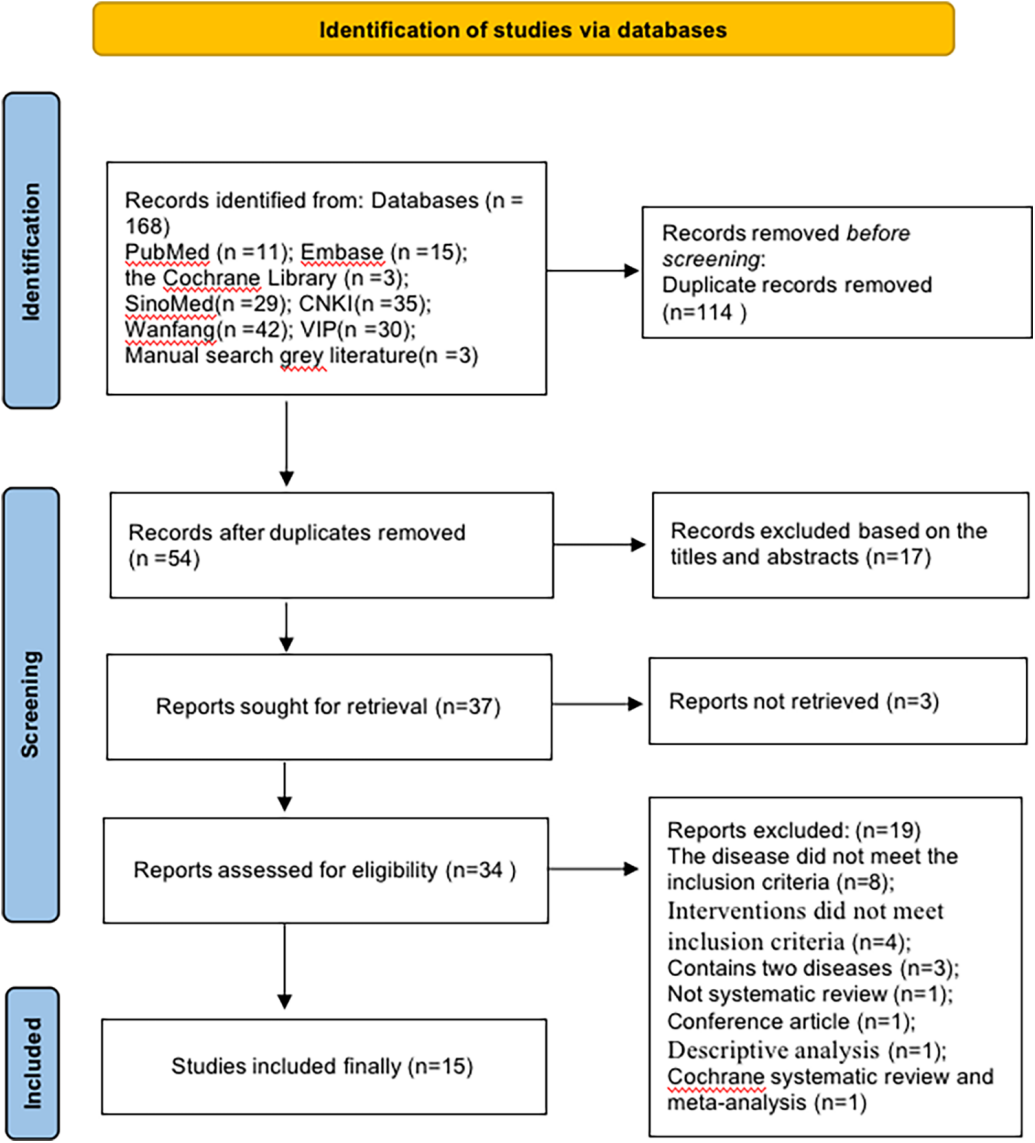
The detailed flow chart.

We retrieved 168 related SRs from seven databases. Of these, we deleted 114 duplicates and then screened 34 studies, followed by a full-text evaluation. Finally, we included 15 SRs. The detailed flow chart is shown in Figure 1. The list of exclusions and reasons are shown in the Supplementary Material of excluded list.

### 3.2 Characteristics of included SRs

The 15 SRs (14–28) were published from 2007 to 202, four SRs (25–28) were published in English, and the other 11 SRs (14–24) were in Chinese. The SRs comprised a total of 329 RCTs and 34,398 subjects. Each SR included 9–78 RCTs, including unstable angina pectoris (14–16, 28), coronary heart disease (17, 18, 27)_,_ and angina pectoris (19–26). Eight SRs defined the diagnostic criteria: three SRs (17, 22, 25) adopted the WHO diagnostic criteria for angina pectoris of CHD, two SRs (16, 27) adopted the Nomenclature and Diagnostic Criteria for Ischemic Heart Disease developed by WHO, one SR (14) adopted the Guidelines for Clinical Research on Drugs in the Cardiovascular System, one SR (23) adopted the International Society of Cardiology and Association of Diagnostic Criteria for Coronary Heart Disease Angina Pectoris and one SR (28) adopted the International Diagnostic Guidelines. The control group usually received conventional therapy, and the treatment group included TXLC or TXLC combined with conventional therapy. The duration was usually 4–8 weeks. Adverse events were reported in 11 SRs (14, 17–20, 22–26, 28), no adverse events were observed in 3 SRs (15, 16, 19), and adverse events were not mentioned in the remaining SRs (27). Regarding the methodological quality assessment, five SRs (14, 19, 21, 22, 25) used the Jadad scale, two SRs (18, 24) did not report this information, and the remaining eight SRs (15–17, 20, 23, 26–28) used the Cochrane Collaboration's RoB assessment tool. The basic characteristics of the literature are shown in Table 1.

**Table 1:** The basic characteristics of the literature.

Author, year (Country)	Trials (subjects)	Diagnostic criteria	Treatment intervention	Control intervention	Adverse reaction	Primary outcomes	Methodological evaluation tool	Main conclusion
Xu GL et al. (14) (Chinese)	15 (727/653)	①	TXLC + CT	CT	①	①②	Jadad	TXLC was superior to CT in relieving AP and improving electrocardiogram.
Yang J et al. (15) (Chinese)	30 (1,188/1,107)	Unclear	TXLC + CT	CT	None	①②③④⑤⑥⑦⑧⑨⑩⑪	Cochrane	The effect of TXLC on UAP was definite.
Wu XQ et al. (16) (Chinese)	14 (661/605)	③	TXLC + CT	CT	None	①②	Cochrane	TXLC combined with CT improved clinical symptoms and electrocardiogram of UAP was better than CT.
Zhou ZR et al. (17) (Chinese)	13 (797/699)	②	TXLC	CT	①②③	①	Cochrane	TXLC was superior to CT in treating AP of CHD.
He HZ et al. (18) (Chinese)	19 (1,005/869)	Unclear	TXLC	CT	①②③④	①	Unclear	TXLC had better clinical efficacy and electrocardiogram efficacy than CT.
Hao CH et al. (19) (Chinese)	20 (1,198/1,026)	Unclear	TXLC	CT	①	①②	Jadad	TXLC was effective and safe in treating angina pectoris.
Wang Y et al. (20) (Chinese)	21 (1,157/1,030)	Unclear	TXLC + CT	CT	①③	①	Cochrane	TXLC combined with CT is more efficient than CT.
Xu GL et al. (21) (Chinese)	9 (570/492)	Unclear	TXLC	CT	None	①②	Jadad	TXLC was better than CT in relieving AP and improving electrocardiogram.
Peng R et al. (22) (Chinese)	10 (434/433)	②	TXLC + CT	CT	①④⑤⑥⑦	①②	Jadad	TXLC combined with CT could effectively improve AP
Chen J et al. (23) (Chinese)	12 (685/460)	④	TXLC	Isosorbide dinitrate	①②③	①②	Cochrane	TXLC is more effective than isosorbide nitrate in treating AP of CHD, and has higher safety.
Fan R et al. (24) (Chinese)	11 (645/617)	Unclear	TXLC + CT	CT	①	①②	Unclear	TXLC combined with CT was better than CT
Jia YL et al. (25) (English)	20 (1,062/874)	②	TXLC	Isosorbide dinitrate	①②③④	①	Jadad	TXLC combined with CT was better than CT
Jia YL et al. (26) (English)	78 (3,444/3,980)	Unclear	TXLC	Betloc	①②③	①	Cochrane	TXLC combined with CT was better than CT
Liu Q et al. (27) (English)	15 (789/789)	③	TXLC + Atorvastatin	Atorvastatin	Unclear	④⑤⑥⑦⑧⑨⑩⑪	Cochrane	XLC combined with atorvastatin was more effective in treating CHD.
Li PQ et al. (28) (English)	42 (2,867/2,554)	⑤	TXLC + CT	CT	①⑤⑧⑨	①③⑥	Cochrane	TXLC is effective and safe for AP.

Diagnostic criteria: ① Guidelines for Clinical Research on Drugs in the Cardiovascular System. ② WHO diagnostic criteria for coronary heart disease angina pectoris. ③ Nomenclature and Diagnostic Criteria for Ischemic Heart Disease developed by WHO. ④ International Society of Cardiology and Association of Diagnostic Criteria for coronary heart disease angina pectoris. ⑤ International diagnostic guidelines are acceptable.

Adverse reaction: ① Digestive reaction. ② Headache. ③Flushing. ④ Dizziness. ⑤ Bleeding. ⑥ Palpitation. ⑦ Chest tightness. ⑧ Hypotension. ⑨ Bradycardia.

Primary outcomes: ① ECG improvement. ② Angina symptoms relief. ③ Incidence of cardiovascular events. ④ Angina attack frequency. ⑤ Duration of angina. ⑥ Hypersensitivity-CRP. ⑦ Total cholesterol. ⑧ Triglyceride. ⑨ High-density lipoprotein cholesterol. ⑩ Low-density lipoprotein cholesterol. ⑪ Adverse reaction.

AP, angina pectoris; UAP, unstable angina pectoris; CHD, coronary heart disease; I, intervention; C, comparison; d, day; w, week; y, year; TXLC, tongxinluo capsule; CT, conventional therapy.9.

### 3.3 Methodological assessment

We used AMSTAR 2 to assess the methodological quality of the 15 SRs. Only one SR had high methodological quality, and in the remaining SRs, the methodological quality could have been much higher. Among the essential items, only two SRs reported the research protocol [item 2 (25, 28)], and only one SR reported the exclusion list [item 7 (28)]. All SRs reported in detail (100%) their database searches (entry 4), fair use of the risk of bias assessment tools (item 9), and risk of bias assessment (item 13). 86.67% of SRs adequately reported on the data synthesis (item 11), and 56.25% of SRs thoroughly investigated and discussed the possible influence of publication bias on the research results (item 15). Among the non-critical items, all SRs provided reasonable information on the inclusion criteria (item 1), literature screening and data extraction (items 5 and 6), and essential characteristics (item 8); moreover, 83.3% of SRs provided a heterogeneity discussion (item 14). However, only 13.3% of SRs reported the reasons for the inclusion of RCTs (item 3), funding sources (item 10), and conflicts of interest (item 16). The detailed results are shown in Table 2.

**Table 2:** The detailed results of AMSTAR-2.

References	AMSTAR-2																Quality
	Item1	Item2	Item3	Item4	Item5	Item6	Item7	Item8	Item9	Item10	Item11	Item12	Item13	Item14	Item15	Item16	
Xu GL et al. (14)	Y	**N**	N	PY	Y	Y	**N**	PY	**Y**	N	**Y**	N	**Y**	Y	**N**	N	Critically low
Yang J et al. (15)	Y	**N**	N	PY	Y	Y	**N**	PY	**Y**	Y	**Y**	Y	**Y**	Y	**Y**	Y	Critically low
Wu XQ et al. (16)	Y	**N**	N	PY	Y	Y	**N**	PY	**PY**	N	**Y**	Y	**Y**	Y	**Y**	N	Critically low
Zhou ZR et al. (17)	Y	**N**	N	PY	Y	Y	**N**	Y	**Y**	N	**Y**	N	**Y**	Y	**Y**	N	Critically low
He HZ et al. (18)	Y	**N**	Y	Y	Y	Y	**N**	PY	**Y**	N	**Y**	Y	**Y**	Y	**Y**	N	Critically low
Hao CH et al. (19)	Y	**N**	N	PY	Y	Y	**N**	PY	**PY**	N	**Y**	Y	**Y**	Y	**Y**	N	Critically low
Wang Y et al. (20)	Y	**N**	N	PY	Y	Y	**N**	PY	**Y**	N	**Y**	N	**Y**	N	**N**	N	Critically low
Xu GL et al. (21)	Y	**N**	N	PY	Y	Y	**N**	PY	**Y**	N	**Y**	N	**Y**	Y	**N**	N	Critically low
Peng R et al. (22)	Y	**N**	Y	PY	Y	Y	**N**	PY	**Y**	N	**Y**	N	**Y**	Y	**N**	N	Critically low
Chen J et al. (23)	Y	**N**	N	PY	Y	Y	**N**	PY	**Y**	N	**Y**	Y	**Y**	Y	**Y**	N	Critically low
Fan R et al. (24)	Y	**N**	N	PY	Y	Y	**N**	PY	**Y**	N	**Y**	Y	**Y**	Y	**Y**	N	Critically low
Jia YL et al. (25)	Y	**Y**	N	PY	Y	Y	**N**	PY	**Y**	N	**N**	N	**Y**	Y	**N**	N	Critically low
Jia YL et al. (26)	Y	**N**	N	PY	Y	Y	**N**	PY	**Y**	N	**N**	N	**Y**	Y	**N**	N	Critically low
Liu Q et al. (27)	Y	**N**	N	PY	Y	Y	**N**	PY	**Y**	N	**Y**	Y	**Y**	Y	**Y**	Y	Critically low
Li PQ et al. (28)	Y	**Y**	N	PY	Y	Y	**Y**	PY	**Y**	Y	**Y**	Y	**Y**	Y	**Y**	Y	High
Y + PY/total (%)	100	**13.3**	13.3	100	100	100	**6.67**	100	**100**	13.3	**86.67**	53.3	**100**	83.3	**60**	13.3	

Y, yes; PY, partial yes; N, no.

### 3.4 RoB

**Figure 2:**
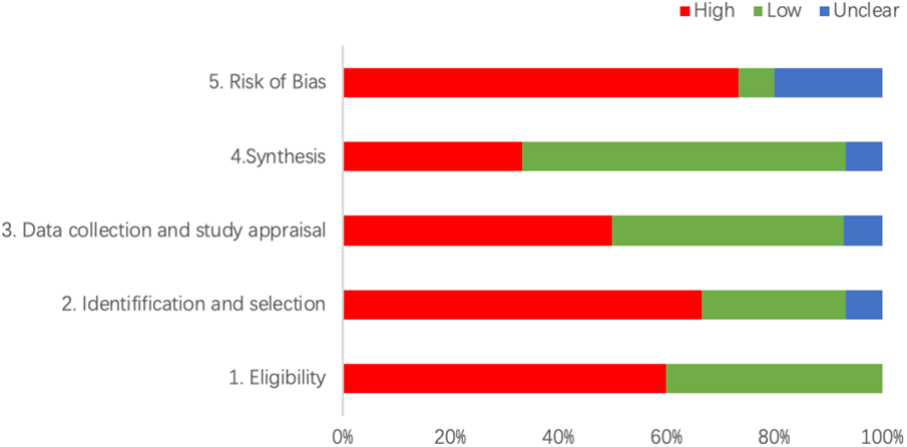
The detailed results of RoB.

The combined results show that only 1 SR had a low risk, 3 SRs (20%) had an unclear risk, and the remaining 11 SRs (73.33%) had a high risk. The detailed results are shown in Figure 2 and Table 3.

**Table 3:** The detailed results of RoB.

Reference	Study eligibility criteria	Identifification and selection of studies	Data collection and study appraisal	Synthesis and fifindings	Risk of bias in the review
Xu GL et al. (14)	high risk	low risk	low risk	low risk	high risk
Yang J et al. (15)	high risk	high risk	high risk	low risk	high risk
Wu XQ et al. (16)	high risk	high risk	high risk	high risk	high risk
Zhou ZR et al. (17)	low risk	unclear risk	low risk	low risk	unclear risk
He HZ et al. (18)	low risk	high risk	low risk	low risk	high risk
Hao CH et al. (19)	high risk	high risk	high risk	low risk	high risk
Wang Y et al. (20)	high risk	high risk	high risk	low risk	high risk
Xu GL et al. (21)	high risk	low risk	high risk	unclear risk	unclear risk
Peng R et al. (22)	high risk	high risk	high risk	high risk	high risk
Chen J et al. (23)	high risk	high risk	high risk	high risk	high risk
Fan R et al. (24)	low risk	low risk	unclear risk	high risk	unclear risk
Jia YL et al. (25)	low risk	low risk	low risk	low risk	low risk
Jia YL et al. (26)	low risk	high risk	high risk	low risk	high risk
Liu Q et al. (27)	high risk	high risk	low risk	high risk	high risk
Li PQ et al. (28)	low risk	high risk	low risk	low risk	high risk

### 3.5 Quality of evidence

We used the GRADE tool to evaluate the evidence quality of 42 outcomes. There were ten outcomes of moderate quality (23.81%), 17 of low quality (40.48%), and 15 of very low quality (35.71%). The main factors influencing demotion were risk of bias, publication bias, imprecision, and inconsistency.

### 3.6 Clinical efficacy of TXLC

Among the 13 SRs that assessed ECG improvement, 6 SRs with outcome measures of moderate quality suggested that TXLC improved cardiac function, but 7 SRs had low-quality evidence. 9 SRs evaluated the time to angina symptom relief, 3 SRs with outcome measures of moderate quality suggested that TXLC shortened the time to angina relief, and the rest were of low/very low quality. In addition, there is limited evidence that TXLC improves the incidence of cardiovascular events, hemorheology, lipid levels, and hs-CRP levels. Thus, TXLC appears to have some efficacy in improving cardiac function and relieving angina pectoris, but the overall efficacy in angina pectoris of CHD should be interpreted with caution.

Eight SRs reported the effectiveness of TXLC in treating AP (14, 19, 20, 22–26), and all of them suggested that the TXLC group had better cardiac function recovery than the control group. Only four SRs had a moderate quality of evidence (19, 20, 24, 25), and the rest of the items were of low quality or very low quality. Five SRs showed that the improvement of angina symptoms in the TXLC group was better than that in the control group (14, 19, 22–24). The evidence quality of only two SRs was moderate (19, 24), and that of the rest was low.

Four SRs reported the effectiveness of TXLC in treating UAP (15, 16, 21, 28). All of them reported electrocardiogram results with TXLC in UAP patients, and the results suggested that TXLC was better than Western medical treatment. Only one SR was rated as moderate quality (15), and the rest of the evidence was low quality. Three SRs suggested that the TXLC group had more significant relief of angina symptoms than the control group (15, 16, 21), and only one had moderate quality of evidence (15). Two SRs suggested that cardiovascular events and hs-CRP incidence were lower in the TXLC group than in the control group. One study reported a significant reduction in the frequency and duration of angina attacks in the TXLC group [SMD = −1.00, 95% CI (−1.58, −0.42), *P* = 0.0007; SMD = −2.25, 95% CI (−3.31, −1.19), *P* < 0.0001]. In addition, there was very-low-quality evidence that TXLC has some efficacy in improving lipid levels in UAP.

### 3.7 Adverse events

12 SRs reported adverse events, mainly gastrointestinal discomfort, such as loss of appetite, nausea, abdominal pain, and abdominal distension, and other adverse events, such as facial flushing, chest tightness, dizziness, headache, and gingival bleeding. A meta-analysis was performed on three SRs for adverse reactions, and two SRs showed no significant difference between the experimental and control groups (15, 27). Another SR (17) showed that the incidence of adverse reactions to TXLC was lower than that of conventional western drugs [RR = 0.33, 95% CI (0.20, 0.53), *P* < 0.00001], but the quality of evidence was low.

### 3.8 Results of the data synthesis and quantitative analysis

**Figure 3:**
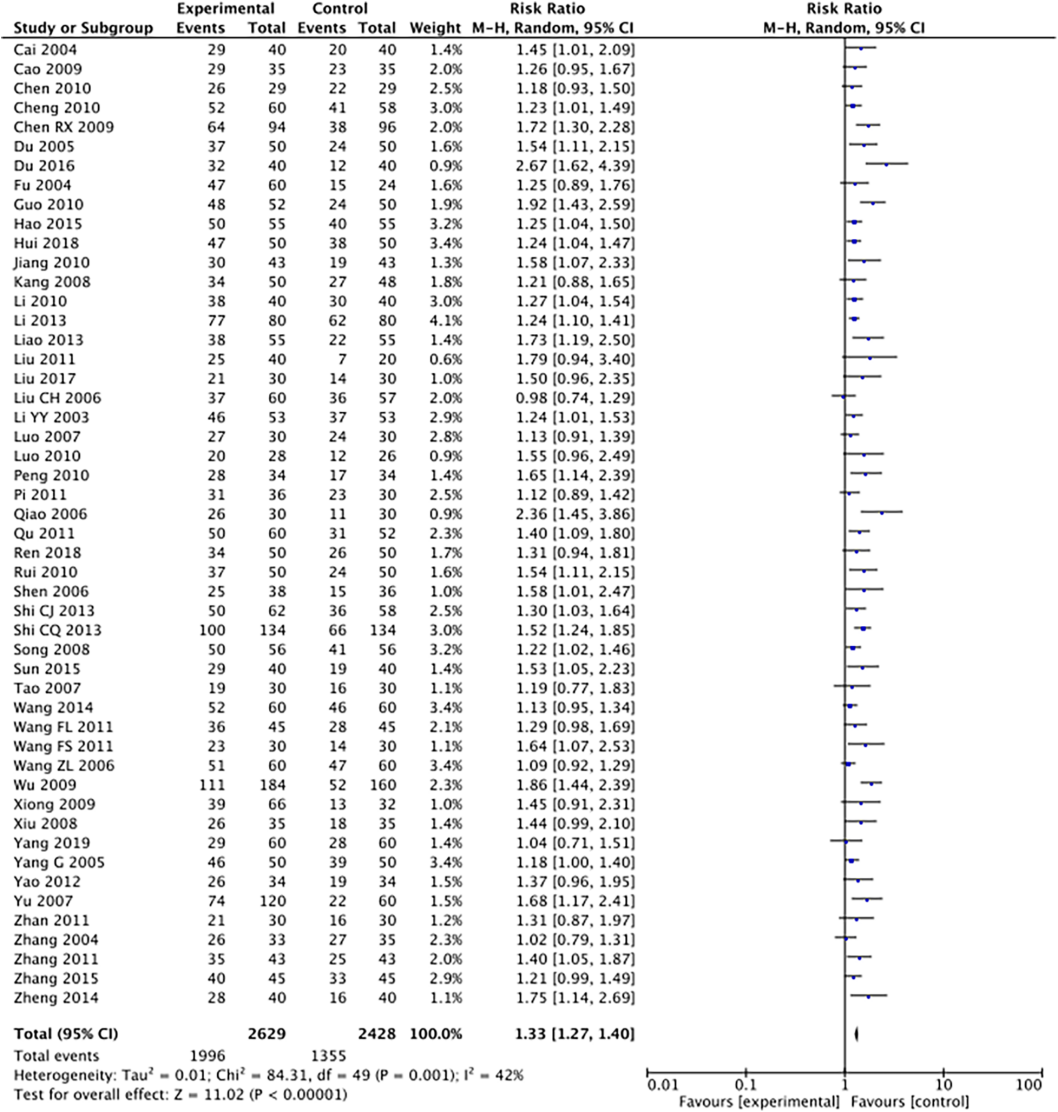
Forest plot of electrocardiography efficacy.

**Figure 4:**
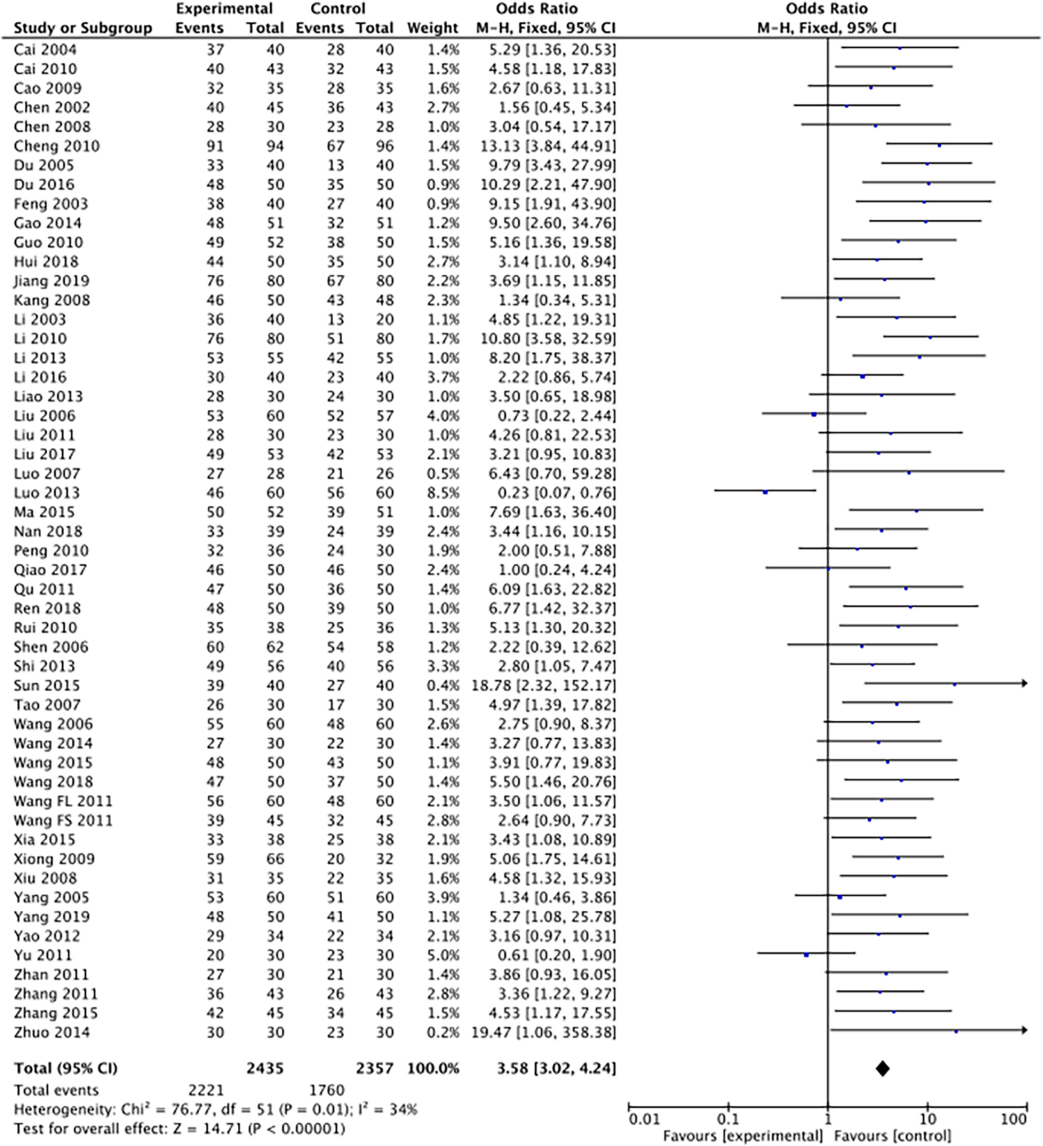
Forest plot of angina pectoris efficacy.

**Figure 5:**
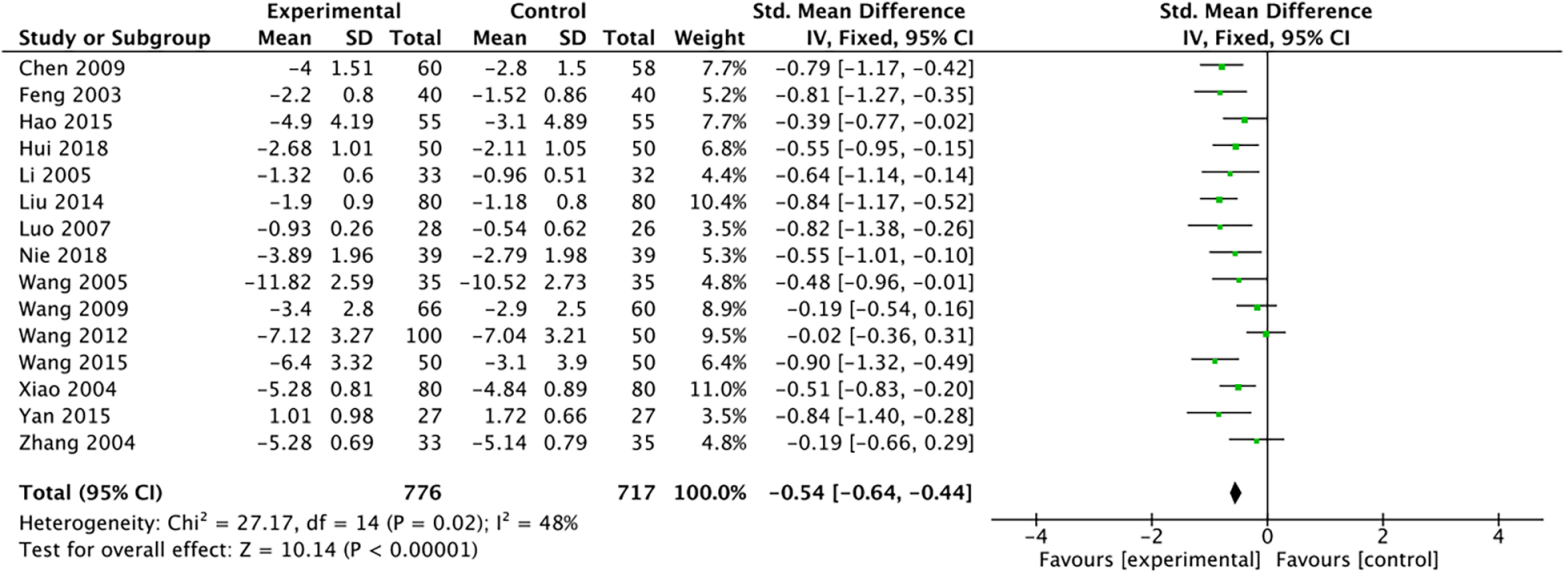
Forest plot of angina attack frequency.

**Figure 6:**
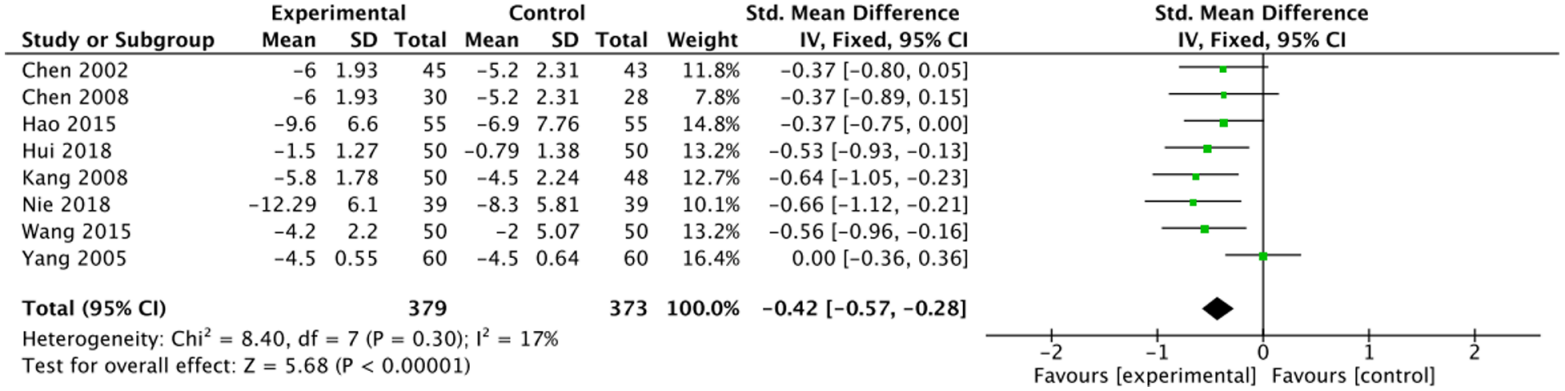
Forest plot of angina duration.

**Figure 7:**
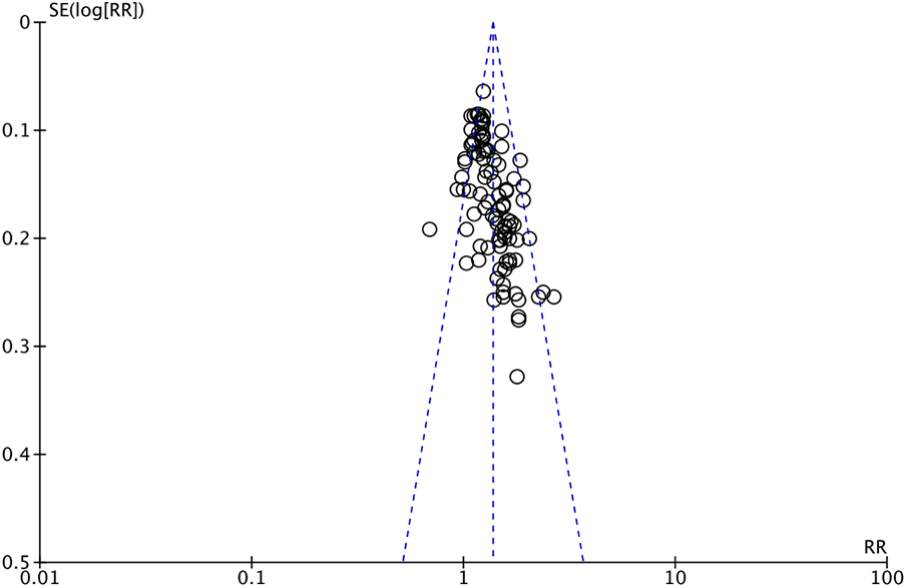
Funnel plot of electrocardiography efficacy.

**Figure 8:**
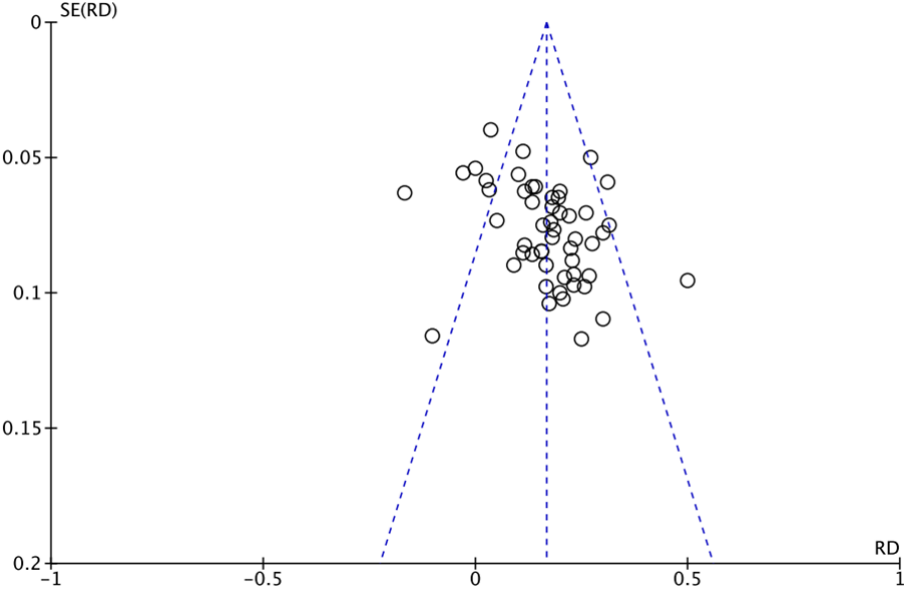
Funnel plot of angina pectoris efficacy.

**Figure 9:**
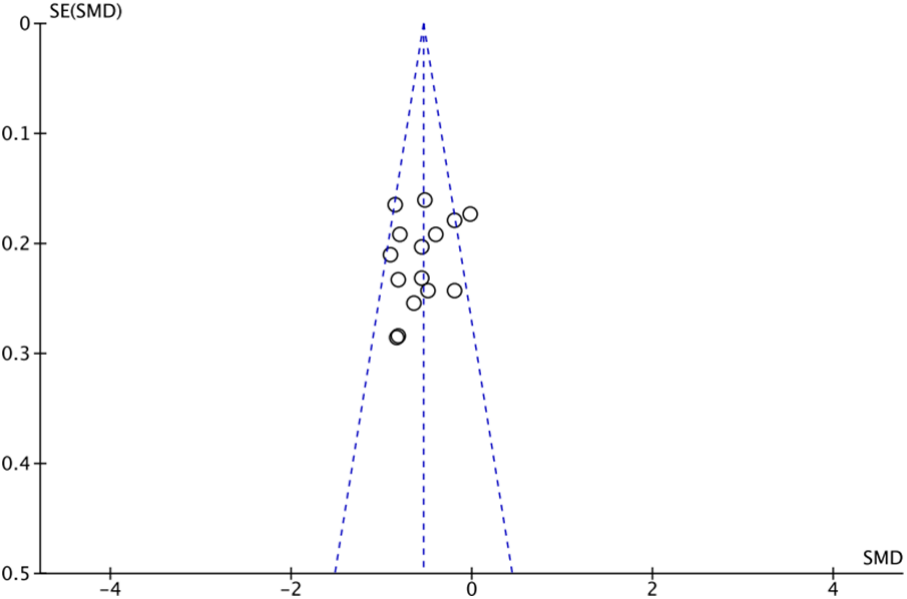
Funnel plot of angina attack frequency.

**Figure 10:**
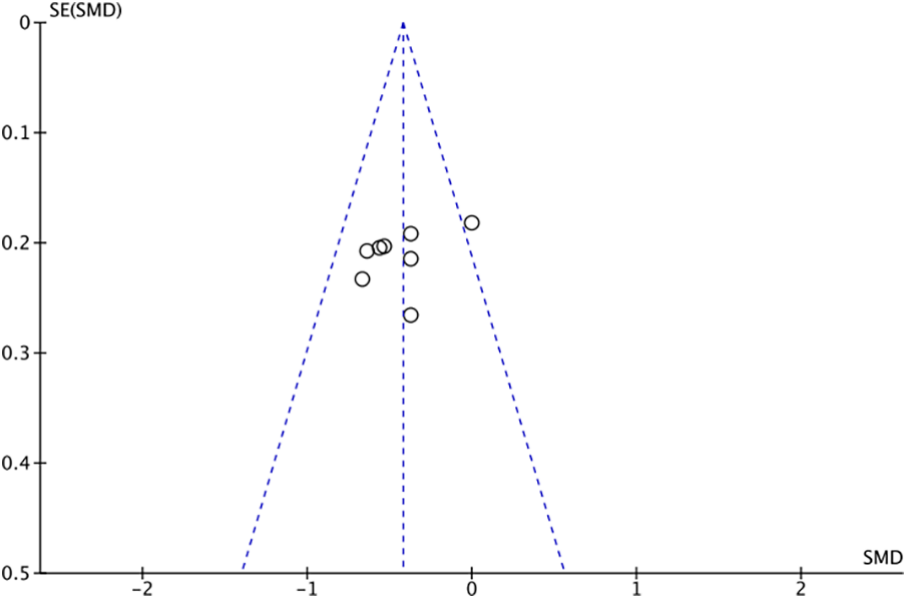
Funnel plot of angina duration.

The outcome index of electrocardiogram efficacy was evaluated in 50 original RCTs screened from 15 SRs. The TXLC group was superior to the traditional therapy group (RR = 1.38, 95% CI: 1.23–1.43, *P* < 0.001), with moderate heterogeneity (*I*^2^ = 42%) and high confidence. According to the outcome index of 52 original RCTs selected from 15 SRs, TXLC combined with Western medicine can effectively improve angina pectoris with high confidence (OR = 3.58, 95% CI: 3.02–4.24, *P* < 0.001, *I*^2^ = 34%). According to the outcome index of 15 original RCTs selected from 15 SRs, TXLC combined with Western medicine was superior to conventional therapy in reducing the frequency of angina attacks (SMD = −0.54, 95% CI: −0.64 to −0.44, *P* < 0.001). Heterogeneity and reliability are moderate (*I*^2^ = 48%). Eight original RCTs selected from 15 SRs were evaluated as an outcome of angina duration. Compared with traditional therapy, the TXLC group can significantly reduce the angina duration (SMD = −0.42, 95% CI: −0.57 to −0.28, *P* < 0.001), with low heterogeneity (*I*^2^ = 17%) and high confidence. Funnel plot analysis of four outcome indexes of RCTs, including electrocardiogram therapy, angina efficacy, angina duration, and angina attack frequency, showed that the visual graph was symmetrical, indicating no obvious publication bias. The detailed results are shown in Figures 3–10.

## 4 Discussion

### 4.1 Summary of the main results

This systematic review summarizes the data from 15 TXLC SRs for angina pectoris in coronary heart disease, including 329 RCTs and 33,417 patients. The results showed that TXLC effectively improved cardiac function and relieved angina pectoris. However, there was insufficient evidence to support the role of TXLC in improving the incidence of cardiovascular events, hemorheology, and lipid levels. The adverse drug effects of TXLC did not appear to be statistically significant compared with those in the control group. The methodological quality of 14 SRs could have been higher, with only 26% of the evidence being of medium quality and over 90% of SRs having unclear or high risk. The results of a single SR confirmed the efficacy of TXLC in relieving angina pectoris of coronary heart disease. However, the results of ROBs evaluation showed that the risk of bias of the SRs was high, which reduced the credibility of their conclusions.

### 4.2 Results-based discussion

Regarding the AMSTER 2 methodological evaluation, 86.67% of SRs did not indicate whether they had pre-registered for the study protocol. A detailed research plan is necessary for a systematic review, and following the plan can reduce the risk of bias in the systematic review process. Almost all SRS did not include a list of exclusions and reasons for exclusion. We could not determine whether researchers purposefully excluded some original studies that affected the SR results, which may have increased selection bias. Nearly 50% of the SR authors did not evaluate the possible impact of the risk of bias in the included studies on the meta-analysis results in detail and did not thoroughly investigate the possibility of publication bias in the quantitative synthesis, which may affect the stability of the results. When conducting a qualitative analysis, the possible influence of the risk of bias on the results of a single study should be discussed, and the publication bias should be reasonably analyzed; for example, the funnel plot and Egger's test should be used to evaluate the publication bias and sensitivity analysis should be used to verify the stability of the results.

Regarding the GRADE evidence quality assessment, only 23.81% of the outcome measures were rated as medium quality. Because of the reasons for the degradation of evidence quality, we summarize the following points: First, the risk of bias was high, mainly due to the methodological quality of the included original research, including various biases in the design, implementation, and measurement of the study. Second, the publication bias was high. The number of RCTs was small, the results were positive, or the funnel plot was asymmetric, which may lead to the biased publication of the outcome indicators. Publication bias is used to determine whether the inclusion of relevant literature meeting the inclusion criteria of a systematic review is comprehensive (such as whether to search grey literature, search research trials, impose language or database restrictions Etc). Commonly used test methods include the funnel plot and Begg and Egger's tests. Third was imprecision, as most of the results of this performance evaluation were degraded due to the small sample size or the small number of RCTs, which resulted in a wide pooled 95% confidence interval. Last is inconsistency. Inconsistency refers to the differences among the original studies included in the systematic review, mainly manifested in the poor overlap of credibility intervals between different studies, significant heterogeneity of pooled results (*I*^2 ^> 50%), or the failure of researchers to reasonably explain results with high heterogeneity.

### 4.3 Pharmacological effects of TXLC

TXLC is composed of *Panax ginseng* C.A. Mey (Ren Shen), *Hirudo nipponica* Whitman (Shui Zhi), *Buthus martensii* Karsch (Quan Xie), *Paeonia lactiflora* Pall. (Chi Shao), *Cicadae periostracum* (Chan Tui), *Scolopendra subspinipes mutilans* L. Koch (Wu Gong), *Eupolyphaga sinensis* Walker (Tu Bie Chong), *Santalum album* L. (Tan Xiang), *Dalbergia odorifera* T.C. Chen (Jiang Xiang), *Boswellia carterii* Birdw. (Ru Xiang), *Ziziphus jujuba* Mill. (Suan Zao Ren), and *Cinnamomum camphora* (L.) J. Presl (Bing Pian). Research shows TXLC can improve plaque stability and reduce inflammation in atherosclerotic rabbits, which may be achieved by modulating intestinal flora and intestinal metabolism (29). Vitro experiments showed that TXLC significantly reduced the extent of injury to Major cardiac adverse events by oxidizing Low-density lipoprotein (30).

Ginsenoside Rg1, the main component of Ren Shen, can inhibit myocardial apoptosis in rats with coronary heart disease by improving the scavenging capacity of oxygen free radicals and inhibiting the expression of inflammatory factors, and the myocardial protection is related to the intervention dose (31). Ginseng polysaccharide (GPS) is a bioactive ingredient with various immunomodulatory, antioxidant, and anticancer functions. GPS could improve the blood lipid abnormality in rats with coronary heart disease, had the function of anti-oxidation and anti-tumor, and had a strong protective effect on mitochondria (32). On the other hand, animal experiments have shown that the volatile oil of the Chuanxiong (Chuanxiong Rhizoma)-Suhexiang (Styrax)-Bingpian (Borneolum) formula can improve EF, FS, and other indices of myocardial damage in a rat model, thus relieving myocardial damage caused by heart hyperactivity, improving cardiac function, and protecting against myocardial damage (33).

Coronary heart disease is mainly caused by hyperlipidemia and hypercholesterolemia due to poor diet quality, too little exercise, and other life factors, which lead to the formation of coronary atherosclerotic plaques, resulting in narrowing or even obstruction of coronary artery vascular channels. In addition, platelet aggregation, inflammation, and oxidative stress will further damage vascular endothelial cells, promote thrombosis, and accelerate coronary atherosclerosis (34). Furthermore, the enrichment results for TXLC-related pathways were mainly related to lipid metabolism, inflammatory response, vasodilation, vascular endothelial cell protection, and cardiomyocyte protection. Toll-like receptors, chemokines, MAPK, TNF, JAK-Stat, and other signaling pathways are related to inflammation (35), while the insulin signaling pathway is related to lipid metabolism. Vascular endothelial growth factor, p53, and the ErbB signaling pathway are related to angiogenesis and apoptosis (36).

In contrast, Fc epsilon RI and the calcium signaling pathway are related to cardiac function, vasodilation, and platelet aggregation (36), and each target and pathway plays a synergistic role. These results indicate that TXLC could act on multiple targets, showing the characteristics of multi-component, multi-target, and integrated regulation of a traditional Chinese medicine compound.

### 4.4 Advantages and limitations

In recent years, with the increase in SRs of TXLC in treating coronary heart disease angina pectoris, their quality has become controversial. Systematic overviews are a new approach to collecting different SR data sets, reassessing methodological quality, and synthesizing individual data. To our knowledge, this is the first systematic overview of TXLC for CHD angina. All SRs were rigorously assessed for methodological quality, risk of bias, and quality of evidence, and two independent reviewers performed all searches and evaluations to obtain reliable results. However, this study also has some limitations, including the limitations of the overview itself and the shortcomings of SRs. First, the system overview can only provide a quantitative synthesis and descriptive data analysis. Secondly, the methodological quality of most SRs could be higher, and the evidence quality of the results could be better, which may be related to the quality of the original research. In addition, due to the limited number of original studies, we needed more advanced data for subgroup analyses and to assess long-term efficacy. Literature reporting following the PRISMA guidelines for SR is also necessary. Finally, TXLC contains animal medicine, which is why most Western countries do not allow similar drugs for clinical use. Differences between formulations and treatment batches are an inevitable consequence of the nature of traditional Chinese medicine, although the Chinese government also sets limits for acceptable differences. These differences may contribute to any heterogeneity between the findings of different studies. In addition, it must be acknowledged that the holistic treatment philosophy of TCM differs from that of Western medicine.

For **Original Research Articles, Clinical Trial Articles**, and **Technology Reports** the introduction.

For more examples of citing other documents and general questions regarding reference style, please refer to the Chicago Manual of Style.

### 4.5 Health, physics and mathematics references

For articles submitted in the domain of Health or the journals Frontiers in Physics and Frontiers in Applied Mathematics and Statistics please apply the Vancouver system for in-text citations.

In-text citations should be numbered consecutively in order of appearance in the text – identified by Arabic numerals in the parenthesis (square parenthesis for Physics and Mathematics).

For some examples please click here.

For more examples of citing other documents and general questions regarding reference style, please refer to Citing Medicine.
