# Supplementary material for: Efficacy and safety of tongxinluo capsule for angina pectoris of coronary heart disease: an overview of systematic reviews and meta-analysis
Source: Front Cardiovasc Med. 2024 Feb 13;11:1229299. doi: 10.3389/fcvm.2024.1229299 (PMC10896841; doi:10.3389/fcvm.2024.1229299)
Supplement: Supplementary file 1 [file Table4.doc]

| Included studies | Outcomes | Number of RCTs (participants) | Certainty assessment | | | | | Effect estimate (95% CI) | *P*-value | Quality of evidence |
| --- | --- | --- | --- | --- | --- | --- | --- | --- | --- | --- |
|  |  |  | Risk of bias | Inconsistency | Indirectness | Imprecision | Publication bias |  |  |  |
| Xu GL et al. (14) | ② | 15 (739/668) | -1 | 0 | 0 | 0 | -1 | OR 6.43 (4.75, 8.71) | *P* < 0.00001 | Low |
|  | ① | 15 (739/668) | -1 | 0 | 0 | 0 | -1 | MD 2.64 (1.78, 3.50) | *P* < 0.00001 | Low |
| Yang J et al. (15) | ③ | 4 (169/126) | -1 | 0 | 0 | 0 | -1 | SMD 0.28 (0.18, 0.45) | *P*＜0.00001 | Low |
|  | ② | 25 (1050/952) | -1 | 0 | 0 | 0 | 0 | SMD 2.46 (1.96, 3.10) | *P*＜0.00001 | Moderate |
|  | ④ | 9 (368/369) | -1 | -1 | 0 | 0 | 0 | SMD -1.00 (-1.58, -0.42) | *P*＝0.0007 | Low |
|  | ⑤ | 5 (189/189) | -1 | -1 | 0 | -1 | -1 | SMD -2.2(-3.31, -1.19) | *P*＜0.0001 | Very Low |
|  | ① | 11 (407/365) | -1 | 0 | 0 | 0 | 0 | OR 2.25 (1.64, 3.09) | *P*＜0.00001 | Moderate |
|  | ⑦ | 6 (256/253) | -1 | -1 | 0 | -1 | -1 | SMD -2.78 (-3.95, -1.62) | *P*＜0.00001 | Very Low |
|  | ⑧ | 9 (375/340) | -1 | -1 | 0 | -1 | 0 | SMD -1.60 (-2.15, -1.05) | *P*＜0.00001 | Very Low |
|  | ⑨ | 9 (375/340) | -1 | -1 | 0 | -1 | 0 | SMD -1.30 (-1.92, -0.68) | *P*＜0.0001 | Very Low |
|  | ⑩ | 8 (330/295) | -1 | -1 | 0 | -1 | -1 | SMD 0.23 (0.05, 0.40) | *P* = 0.009 | Very Low |
|  | ⑪ | 8 (296/261) | -1 | -1 | 0 | -1 | -1 | SMD -0.83 (-1.35, -0.32) | *P*＝0.002 | Very Low |
|  | ⑥ | 5 (214/215) | -1 | 0 | 0 | -1 | -1 | SMD 0.87 (0.48, 1.55) | *P* = 0.63 | Very Low |
| Wu XQ et al. (16) | ② | 14 (661/605) | -1 | 0 | 0 | 0 | -1 | RR 1.26 (1.19, 1.34) | *P*＜0.00001 | Low |
|  | ① | 13 (601/545） | -1 | 0 | 0 | 0 | -1 | RR 1.31 (1.20, 1.42) | *P*＜0.00001 | Low |
| Zhou ZR et al. (17) | ① | 13 (797/699) | -1 | 0 | 0 | 0 | -1 | RR 0.56 (0.49, 0.64) | *P* < 0.00001 | Low |
|  | ⑥ | 10 (545/477) | -1 | 0 | 0 | 0 | -1 | RR 0.33 (0.20, 0.53) | *P* < 0.00001 | Low |
| He HZ et al. (18) | ① | 17 (954/819) | -1 | 0 | 0 | 0 | 0 | OR 2.38 (1.83, 3.09) | *P* < 0.00001 | Moderate |
| Hao CH et al. (19) | ② | 20 (1193/1024) | -1 | 0 | 0 | 0 | 0 | OR 3.66 (2.91, 4.60) | *P*＜0.00001 | Moderate |
|  | ① | 18 (983/734) | -1 | 0 | 0 | 0 | 0 | OR 2.58 (2.12, 3.15) | *P*＜0.00001 | Moderate |
| Wang Y et al. (20) | ① | 21 (1157/1030) | -1 | 0 | 0 | 0 | 0 | OR 2.00 (1.64, 2.43) | *P*< 0.00001 | Moderate |
| Xu GL et al. (21) | ② | 9 (570/488) | -1 | 0 | 0 | 0 | -1 | OR 5.61 (399, 7.89) | *P* < 0.00001 | Low |
|  | ① | 9 (570/488) | -1 | 0 | 0 | 0 | -1 | OR 2.45 (1.90, 3.16) | *P* < 0.00001 | Low |
| Peng R et al. (22) | ② | 10 (434/433) | -1 | 0 | 0 | 0 | -1 | OR 4.01 (2.68, 5.99) | *P* < 0.00001 | Low |
|  | ① | 3 (115/115) | -1 | 0 | 0 | -1 | -1 | OR 1.67 (0.94, 2.98) | *P* = 0.08 | Very Low |
| Chen J et al. (23) | ② | 12 (606/530) | -1 | 0 | 0 | 0 | -1 | OR 2.74 (1.95, 3.85) | *P*＜0.00001 | Low |
|  | ① | 12 (606/530) | -1 | 0 | 0 | 0 | -1 | OR 2.65 (2.05, 3.42) | *P*＜0.00001 | Low |
| Fan R et al. (24) | ② | 11 (645/617) | -1 | 0 | 0 | 0 | 0 | RR 1.25 (1.18, 1.32) | *P* < 0.00001 | Moderate |
|  | ① | 9 (560/534) | -1 | 0 | 0 | 0 | 0 | RR 1.40 (1.28, 1.54) | *P* < 0.00001 | Moderate |
| Jia YL et al. (25) | ① | 18 (987/806) | -1 | 0 | 0 | 0 | 0 | OR 2.38 (1.84, 3.09) | *P* < 0.00001 | Moderate |
| Jia YL et al. (26) | ① | 47 (2063/1612) | -1 | 0 | 0 | 0 | -1 | OR 2.63 (2.29, 3.02) | *P* < 0.0001 | Low |
| Liu Q et al. (27) | ⑧ | 9 (475/485) | -1 | 0 | 0 | 0 | 0 | MD -1.21 (-1.53, -0.89) | *P*＜0.00001 | Moderate |
|  | ⑨ | 9 (426/426) | -1 | 0 | 0 | 0 | -1 | MD -0.73 (-0.81, -0.65) | *P*＜0.00001 | Low |
|  | ⑩ | 6 (321/322) | -1 | 0 | 0 | -1 | -1 | MD 0.27 (0.23, 0.31) | *P*＜0.00001 | Very Low |
|  | ⑪ | 8 (383/382) | -1 | 0 | 0 | -1 | -1 | MD -0.72 (-0.80, -0.64) | *P*＜0.00001 | Very Low |
|  | ⑦ | 7 (345/345) | -1 | -1 | 0 | -1 | 0 | SMD -2.06 (-2.56, -1.57) | *P*＜0.00001 | Low |
|  | ④ | 5 (265/265) | -1 | -1 | 0 | -1 | -1 | SMD -1.41 (-1.97, -0.85) | *P*＜0.00001 | Very Low |
|  | ⑤ | 4 (186/186) | -1 | 0 | 0 | -1 | -1 | MD -2.30 (-3.39, -1.21) | *P*＜0.0001 | Very Low |
|  | ⑥ | 6 (347/348) | -1 | 0 | 0 | -1 | -1 | RR 0.84 (0.51, 1.39) | *P*= 0.50 | Very Low |
| Li PQ et al. (28) | ③ | 6 (490/419) | -1 | 0 | 0 | -1 | -1 | RR 0.29 (0.19, 0.45) | *P*＜0.00001 | Very Low |
|  | ① | 13 (891/749) | -1 | 0 | 0 | 0 | -1 | RR 1.23 (1.16, 1.30) | *P*＜0.00001 | Low |
|  | ⑦ | 4 (260/256) | -1 | -1 | 0 | -1 | -1 | MD -2.86 (-3.73, -1.99) | *P*＜0.00001 | Very Low |

**Outcomes:** ① ECG improvement. ② Angina symptoms relief. ③ Incidence of cardiovascular events. ④ Angina attack frequency. ⑤ Duration of angina. ⑥ Adverse reaction. ⑦Hypersensitivity-CRP. ⑧ Total cholesterol. ⑨ Triglyceride. ⑩ High-density lipoprotein cholesterol. ⑪ Low-density lipoprotein cholesterol.
