# Supplementary material for: Efficacy and safety of tongxinluo capsule for angina pectoris of coronary heart disease: an overview of systematic reviews and meta-analysis
Source: Front Cardiovasc Med. 2024 Feb 13;11:1229299. doi: 10.3389/fcvm.2024.1229299 (PMC10896841; doi:10.3389/fcvm.2024.1229299)
Supplement: Supplementary file 2 [file Table5.doc]

PubMed database

| #1 | ((((((((Angina Pectoris[MeSH Terms]) OR (Coronary Disease[MeSH Terms])) OR (Coronary heart disease[Text Word])) OR (CHD[Text Word])) OR (Coronary atherosclerotic heart disease[Text Word])) OR (coronary atherosclerotic cardiopathy[Text Word])) OR (angina[Text Word])) OR (stenocardia[Text Word])) OR (angor pectoris[Text Word]) |
| --- | --- |
| #2 | (((((Tong-xin-luo) OR (Tong xin luo)) OR (tong xin luo)) OR (Tongxinluo)) OR (tongxinluo)) OR (tongxinluo capsule)[[All Fields ] |
| #3 | ((systematic review[Text Word]) OR (systematic evaluation[Text Word])) OR (meta analysis[Text Word]) |
| #4 | #1 AND #2 AND #3 |

Embase database

| #1 | 'angina pectoris':ti,ab,kw OR 'coronary disease':ti,ab,kw OR 'coronary heart disease':ti,ab,kw OR chd:ti,ab,kw OR 'coronary atherosclerotic heart disease':ti,ab,kw OR 'coronary atherosclerotic cardiopathy':ti,ab,kw OR angina:ti,ab,kw OR stenocardia:ti,ab,kw OR 'angor pectoris':ti,ab,kw |
| --- | --- |
| #2 | 'tong xin luo':ti,ab,kw OR tongxinluo:ti,ab,kw OR 'tongxinluo capsule':ti,ab,kw |
| #3 | 'systematic review':ti,ab,kw OR 'systematic evaluation':ti,ab,kw OR 'meta analysis':ti,ab,kw |
| #4 | #1 and #2 and #3 |

Cochrane library database

| #1 | (Tong-xin-luo):ti,ab,kw |
| --- | --- |
| #2 | (Tong xin luo):ti,ab,kw |
| #3 | (tong xin luo):ti,ab,kw |
| #4 | (Tongxinluo):ti,ab,kw |
| #5 | (tongxinluo):ti,ab,kw |
| #6 | (tongxinluo capsule):ti,ab,kw |
| #7 | #1 OR #2 OR #3 OR #4 OR #5 OR #6 |
| #8 | Angina Pectoris: MeSH |
| #9 | Coronary Disease:MeSH |
| #10 | (Coronary heart disease):ti,ab,kw |
| #11 | (CHD):ti,ab,kw |
| #12 | (Coronary atherosclerotic heart disease):ti,ab,kw |
| #13 | (coronary atherosclerotic cardiopathy):ti,ab,kw |
| #14 | (angina):ti,ab,kw |
| #15 | (stenocardia):ti,ab,kw |
| #16 | (angor pectori):ti,ab,kw |
| #17 | #8 OR #9 OR #10 OR #11 OR #12 OR #13 OR #14 OR #15 OR #16 |
| #18 | (systematic review):ti,ab,kw |
| #19 | (systematic evaluation):ti,ab,kw |
| #20 | (meta-analysis):ti,ab,kw |
| #21 | #18 OR #19 OR #20 |
| #22 | #7 AND #17 AND #21 |
