# Supplementary material for: Efficacy and safety of tongxinluo capsule for angina pectoris of coronary heart disease: an overview of systematic reviews and meta-analysis
Source: Front Cardiovasc Med. 2024 Feb 13;11:1229299. doi: 10.3389/fcvm.2024.1229299 (PMC10896841; doi:10.3389/fcvm.2024.1229299)
Supplement: Supplementary file 3 [file Table6.doc]

Excluded list

| Citation | Reason for exclusion |
| --- | --- |
| Li M, Li C, Chen S, Sun Y, Hu J, Zhao C, et al. Potential Effectiveness of Chinese Patent Medicine Tongxinluo Capsule for Secondary Prevention After Acute Myocardial Infarction: A Systematic Review and Meta-Analysis of Randomized Controlled Trials. Front Pharmacol. 2018 Aug 3;9:830. | The disease did not meet the inclusion criteria |
| Mao HM, Liu M, Qu H, Wang LQ, Shi DZ. Tongxinluo Capsule for Cardiac Syndrome X: A Systematic Review and Meta-Analysis. Chin J Integr Med. 2018 Apr;24(4):296-303. | The disease did not meet the inclusion criteria |
| Mao C, Fu XH, Yuan JQ, Yang ZY, Chung VC, Qin Y, et al. Tong-xin-luo capsule for patients with coronary heart disease after percutaneous coronary intervention. Cochrane Database Syst Rev. 2015 May 21;(5):CD010237. | The disease did not meet the inclusion criteria |
| Z Zhou, H Tang, J Li, L Yang, H Hu.[Tongxinluo capsule for coronary heart disease: a systematic review](https://www.cochranelibrary.com/central/doi/10.1002/central/CN-01740954/full).Heart (British Cardiac Society), 2010, 96, A103. | Conference article |
| He Xiu, Lu CY, Li JQ, Jing N, Liu YM. A meta-analysis of tongxinluo capsule in the treatment of coronary heart failure [J]. Chinese patent medicine,2019,41(07):1572-1577. | The disease did not meet the inclusion criteria |
| Wu CX. Meta-analysis of clinical efficacy of Tongxinluo capsule in patients with acute myocardial infarction after reperfusion [D]. Guangxi University of Chinese Medicine,2017. | The disease did not meet the inclusion criteria |
| Bai Y, Che FF, Yi WH, Bai RN, Feng B, Xi RX, et al. Effects of oral Chinese patent medicine on major cardiovascular events after PCI: a meta-analysis [J]. Chinese journal of traditional Chinese medicine,2018,59(12):1024-1030. | The disease did not meet the inclusion criteria |
| Zhang FF, Xie HB. Meta-analysis of the effect of Tongxinluo capsule on angina pectoris after PCI [J]. Chinese Journal of Medical Innovation, 201,18(21):132-138. | The disease did not meet the inclusion criteria |
| Huang CH, Jiang KL, Sun WP, et al. Meta-analysis of the effect of tongxinluo capsule on hemorheology in patients with coronary heart disease [J]. China journal of basic medicine of traditional Chinese medicine,2018,24(07):955-960. | The disease did not meet the inclusion criteria |
| Geng JW, Shi TY, Zhang L, Sun WP, Ji SL, Lin T, et al. A meta-analysis of the efficacy of Tongxinluo capsules combined with statins in the treatment of coronary heart disease with hypertension [J]. Chinese pharmacy,2018,29(19):2694-2698. | Contains two diseases |
| Zhang SJ, Zhao QY, Dai ZX, Wang XZ, Zhao HY, Wei HT, et al. Efficacy and safety of Shensong Yangxin Capsule combined with Tongxinluo Capsule in treatment of coronary heart disease complicated with premature ventricular contractions [J]. Chinese journal of cardiovascular disease,2015,0(10):878-881. | Contains two diseases |
| Geng HJ, Xie YM, Wang ZF. Clinical evaluation of Naoxintong capsule in treatment of angina pectoris of coronary heart disease and cerebral infarction with Qi deficiency and blood stasis syndrome [J]. China Journal of Chinese Materia Medica, 201,46(23):6087-6095. | Not systematic review |
| Geng JW, Shi TY, Zhang L, Sun WP, Ji SL, Lin T, et al. A meta-analysis of the efficacy of Tongxinluo capsules combined with statins in the treatment of coronary heart disease with hypertension [J]. Chinese pharmacy,2018,29(19):2694-2698. | Contains two diseases |
| Lin XD, Tang JM, Yang JY, Zhang L, Cao T, Jiang FB, et al. Meta-analysis of treatment of angina pectoris with tongxinluo combined with betalprok in patients with coronary heart disease [J]. Chinese journal of clinical health,2017,20(02):166-169. | Interventions did not meet inclusion criteria |
| Yang K, Jiang H. A meta-analysis of tongxinluo capsule combined with trimetazidine in the treatment of unstable angina pectoris [J]. Hainan med,2015,26(07):1061-1065. | Interventions did not meet inclusion criteria |
| Jia YL, Zhang SK Bao FF, Huang FY, Liang SW. Systematic evaluation of tongxinluo Capsule and Danshen drop pill in the treatment of coronary heart disease angina pectoris [J]. Chinese journal of evidence-based medicine,2011,11(08):919-931. | Interventions did not meet inclusion criteria |
| Zhong YX. Systematic evaluation of Tongxinluo capsule in the treatment of CAHD [J]. Medical Information,2013(25):471-471. | Descriptive analysis |
| Liu Y, Zhang W, Fan JM. Effect of Tongxinluo capsule on vascular endothelial function in patients with coronary heart disease: a meta-analysis [J]. Chinese Journal of Intractable Diseases,202,21(02):139-144+150. | Interventions did not meet inclusion criteria |
| Wu T, Harrison RA, Chen X, et al. Tongxinluo (Tong xin luo or Tong-xin-luo) capsule for unstable angina pectoris. Cochrane Database Syst Rev. 2006;2006(4):CD004474. | Cochrane systematic review and meta-analysis |
